# Video-based long-term follow up of musician’s dystonia in pianists reveals similar improvements following different treatment strategies: a retrospective observational study

**DOI:** 10.1186/s40734-021-00092-3

**Published:** 2022-01-25

**Authors:** Julius Gründahl, Michael Großbach, Eckart Altenmüller

**Affiliations:** 1 Institut für Musikphysiologie und Musiker-Medizin, Hochschule für Musik, Theater und Medien, Hannover, Germany; 2 Medizinische Hochschule Hannover, Carl-Neuberg-Str. 1, 30625 Hannover, Germany

**Keywords:** Musician’s dystonia, Video rating, Botulinum-toxin, Trihexyphenidyl, Retraining, Interrater reliability, Therapy response

## Abstract

**Background:**

Musician’s Dystonia (MD) by impaired or complete loss of fine motor control in extensively trained movements at the instrument. In pianists, it frequently leads to involuntary flexion or extension of one or more fingers. Treatment remains challenging, although local injections with Botulinum toxin, anticholinergic medication and pedagogical retraining seem to be helpful in individual cases. Evaluation of therapies however is frequently hampered by lack of practicability, or validity. This retrospective observational study aims to assess the long-term development of MD and efficacy of therapies in keyboard players by means of a simple video-rating procedure by informed expert raters.

**Methods:**

Video rating is characterisedwas done by 6 carefully instructed pianists, rating a total of 266 videos from 80 patients, recorded over a period of almost 20 years. These showed the affected hand playing a C-major scale as “regularly” as possible at a moderate tempo on a grand piano. Raters assessed the acoustic irregularity of scale playing and any visible movement impairment on visual analogue scales. Influence of patient-specific factors including applied treatment was estimated in a Bayesian multilevel beta regression.

**Results:**

For ratings of irregularity and impairment intra-rater reliability was strong and inter-rater concordance was moderate. The average estimated improvement across all therapies was 14% in irregularity and 15% in impairment. Highest improvement rates in ratings of irregularity were found after the combined treatment with Botulinum toxin and Trihexyphenidyl (45%) as well as in retraining (29% improvement) as single therapy. In ratings of impairment the highest improvement was shown for retraining in combination with Trihexyphenidyl (36% improvement) as well as retraining as single therapy (23%).

**Conclusions:**

This study provides a new perspective on the course of MD in keyboard players in a larger cohort using methods other than self-report. Video rating of scale-playing was shown to be a reliable and useful method to evaluate MD in keyboard players. Average improvement rates were different to previous studies using patient-subjective questionnaires. Treatment options showed different effects in the two rating criteria, with retraining showing the highest improvement rates in single and combined use.

**Supplementary Information:**

The online version contains supplementary material available at 10.1186/s40734-021-00092-3.

## Background

Musician’s Dystonia (MD), often termed musician’s cramp, is a focal and task-specific form of dystonia affecting voluntary control of muscles or muscle groups used in highly precise and extensively trained movements while playing a musical instrument [1]. Common symptoms are either impaired or complete loss of task-relevant voluntary motor control, leading to stiffness or cramping, the latter resulting in involuntary movements of affected fingers, i.e. flexion or extension [2, 3]. According to rough estimations, 1 % of all professional musicians are affected by MD, yet the dark number is probably higher [2, 4]. MD has been described for almost every instrument group, with keyboard players being the most affected [5].

The pathophysiology of MD is not completely understood at present, but it is probably multifactorial. Most studies of focal dystonia reveal abnormalities in three main areas: a) reduced inhibition in the motor system at cortical, subcortical and spinal levels; b) altered sensory perception and integration; and c) impaired sensory-motor integration. All of these changes are believed to primarily originate from altered synaptic plasticity and dysfunctional brain plasticity [6–8]. Furthermore, overuse, chronic stress and traumatic life experiences might trigger MD, given a genetic susceptibility [9, 10].

Treatment options in MD include in first line symptomatic pharmacological treatment with local injections with Botulinumtoxin. EMG- or ultrasound-guided injection improves motor performance in most patients, however, requires exact identification of the dystonic muscle and regular injections by trained clinicians [11–16]. In oral medication, the anticholinergic drug Trihexyphenidyl has been proven to be effective; nevertheless, frequent side effects such as dry mouth, fatigue and confusion have been reported, and an effect has been shown only for limb dystonia [11, 12]. As a behavioural therapy, pedagogical retraining has been introduced with the aim of restoring the disturbed cortical motor program. To this end, dystonic movements are identified, and then limited to a speed level at which they would not occur and thus compensatory movements are avoided. A correct movement pattern is introduced and the patient’s awareness is trained by the use of visual feedback and body awareness techniques [17]. Such a retraining requires a long therapeutic process and high intrinsic motivation by the patient, but has proven to lead to long-term improvement of symptoms [17–19].

The long-term development of symptoms in MD has been described in several studies, using different assessment tools. Jabusch et al. [11], Schuele et al. [13] and van Vugt et al. [17] have mainly used patient-subjective questionnaires with ordinal scales, reporting an improvement in symptoms in 52, 69 and 82% of the patients, respectively. As an objective evaluation method, MIDI-based scale playing analysis was introduced by Jabusch et al. in 2004 [20] and was additionally used in the study by van Vugt et al., revealing an improvement in 43% of the patients [17].

Although several clinical scales for dystonia rating are available, especially the Burke-Fahn-Marsden Movement Disability Scale and Unified dystonia rating scale (for review see [21]), these scales frequently lack sensitivity in MD-patients, since frequently the only symptom is a minor hyperflexion or extension of one or two digits while playing the instrument. Therefore, Spector and Brandfonbrener developed the more specific Frequency of Abnormal Movements Scale, accounting for the frequency of dystonic flexion and compensatory extension movements while playing the instrument [22]. However, although these objective assessments have been proven useful, they are time consuming, depend on technology and cannot be easily applied to other than pianistic movements. Therefore, there is still a need for standardized rating procedures which are easy to apply, feasible in clinical practice approved in large, controlled studies [23]. In the following, we will present a retrospective, observational study assessing the long-term development of dystonic symptoms in a large cohort of keyboard players. The temporal development of symptoms was evaluated from video snippets recorded at the beginning and during or at the end of the treatment phase, introducing an informed rating procedure by piano-major students using visual analogue scales (VAS). The influence of several covariates, including the administered therapy, on the development of symptoms was evaluated in a Bayesian multilevel beta regression. Comprising 80 patients with clinical examinations performed between 1997 and 2016, it is so far the largest patient cohort with the longest observational period on focal dystonia in keyboard players.

The aim of this study therefore was twofold, namely 1) assess the long-term development and efficacy of therapies in keyboard players suffering from MD based on a large video collection comprising routinely performed videos of scale playing covering up to 15 years of treatment and 2) demonstrate the practicability and appropriateness of a rating procedure by informed and trained student expert raters.

## Methods

As part of the examination at the outpatient clinic of the Institute of Music Physiology and Musicians’ Medicine Hanover over the last 25 years, most of the patients affected by MD were videotaped while playing their instrument. The recordings’ main purpose was to objectively document the progression of the disorder for both clinicians and patients. The camera was usually placed to the right of the keyboard, capturing the patients’ hands and forearms. Patients played up- and downwards C-major scales over two octaves, reportedly a difficult task for pianists with dystonic symptoms [20]. This task requires not only very even and controlled movements of the five fingers, but also the difficult thumb-passage, i.e. the anticipatory flexion in ulnar-radial movements of the thumb under fingers 2–5, with no black keys involved. Since all classically educated pianists have been trained to play this scale as evenly and fast as possible, C-major scale playing has been identified as a highly automated, reliable, specific and sensitive measure of pianists’ skills [24].

Video snippets of C-major scales were collected, and pianists suffering from MD with two or more such videos recorded at different time points were included in the study. All patients gave informed consent for recording and usage of the videos in the present study. Patient features other than hand or forearm were pixelated when visible in the video. Videos in which patients talked whilst playing were excluded. The study was approved by the ethics committee of the Hanover Medical School (Approval number 3690-2017).

### Patient characteristics

Eighty patients (22 female) with a total of 266 videos, (median: 3, range: 2–12), were included in the study. The first video was recorded in September 1997, the last one in March 2016. The median time between the first and the last video for a single patient was 33 months (0–184). Owing to different playing tempi, videos had durations between 5 and 53 s (median 20). 73 patients were pianists, 6 were organists and one patient played harpsichord as their main instrument. All patients were diagnosed with focal dystonia by an experienced clinician and movement disorders specialist (EA), and 64 patients were found to be affected in the right hand and 14 in the left. In 2 patients affected in both hands, we decided to only rate the hand affected first in the course of the disorder. Only two right hand affected patients reported in the follow up additional minor symptoms when writing, with a feeling of tension and discomfort when writing. While at the onset of symptoms patients were a median of 31.5 years old (range: 18–68), by the time of the first recording patients had a median age of 37 years (21–71; two patients’ files did not contain information about the onset of symptoms). The period between first symptoms and the first appointment at the clinic was between one and 480 months (median: 27). Therapy was mainly restricted to Botulinum-Toxin injections (BTX), Trihexiphenidyl (TRHX), retraining (RTR), or a combination thereof: 27 patients solely attended a retraining program, 10 patients only received BTX-injections and six were treated solely with TRHX; nine patients received a combination of BTX and RTR, and seven were administered a combination of either RTR and TRHX, or BTX and TRHX. Thirteen patients received a combination of all three therapies. One patient with only two videos included in the study received no treatment at all between the two appointments. Inclusion in therapy groups was independent from administered dose or treatment duration.

Patients were asked during the examination whether they were aware of relatives suffering from a movement disorder, which was confirmed by 6 patients (7.5%), whereas 57 answered in the negative (71.25%); for 17 patients (21.25%) there was no information available in the patient files. Patient data are also reported in Table 1.

**Table 1:** Clinical characteristics of patients

Number	Gender	Treatment duration	Age 1st symptoms	Time to treatment	Affected side	Affected finger(s)	Therapy	Family history of Dystonia
1	f	44	40	50	right	3	BTX, RTR	n.a.
2	m	68	23	12	right	Wrist, 2	BTX, RTR, TRHX	–
3	m	31	22	24	left	2	BTX	n.a.
4	m	38	20	7	right	3,4	BTX, RTR, TRHX	–
5	f	49	50	20	right	4,5	BTX, RTR, TRHX	–
6	m	10	50	12	right	3	BTX	n.a.
7	m	16	24	1	right	3	RETR	–
8	f	44	24	6	right	2,3	RETR	–
9	m	57	37	60	right	3	BTX, RTR, TRHX	+
10	f	22	52	60	right	4	BTX, RTR	–
11	m	26	33	5	right	4	BTX, RTR, TRHX	–
12	f	84	25	84	right	1,2	RTR	–
13	m	43	47	60	right	2,3,4,5	BTX, RTR	–
14	m	30	22	72	right	4,5	RTR	–
15	m	68	31	4	right	3,4,5	BTX, RTR	–
16	m	42	44	108	right	1,3,4	RTR, TRHX	–
17	f	23	28	12	right	1	RTR	–
18	f	7	28	2	left	4,5	RTR	–
19	m	24	20	132	right	3	BTX, RTR	–
20	m	31	39	264	left	2,3,4	BTX, RTR	–
21	f	46	23	6	left	2	RTR	n.a.
22	m	21	33	156	right	4,5	BTX, TRHX	–
23	m	24	40	12	right	2,3,4	RTR	–
24	m	18	n.a.	n.a.	right	1,2,3,4,5	BTX, RTR	+
25	f	6	30	30	right	n.a.	RTR, TRHX	–
26	m	56	31	120	right	3,4	BTX	–
27	f	8	22	24	right	3,4	RTR	–
28	m	48	27	24	right	3	RTR	–
29	f	25	40	12	right	n.a.	BTX, RTR, TRHX	–
30	m	27	37	24	right	n.a.	RTR	–
31	m	3	29	36	right	3	RTR	–
32	f	32	29	276	right	1,2	RTR	+
33	f	14	24	9	right	4,5	RTR	–
34	m	23	20	48	right	2	RTR, TRHX	+
35	m	133	40	60	right	3	BTX, RTR, TRHX	n.a.
36	m	30	24	2	right	4	RTR	–
37	m	95	27	24	right	3	BTX, RTR, TRHX	–
38	m	99	46	36	right	3	BTX	–
39	m	45	37	8	right	4,5	RTR	–
40	m	103	50	108	right	Wrist	BTX, RTR, TRHX	–
41	f	117	44	96	right	3	BTX, RTR, TRHX	–
42	f	72	22	8	right	Wrist	RTR	–
43	m	83	24	8	right	2	RTR, TRHX	n.a.
44	m	34	68	36	right	3,4	BTX	n.a.
45	m	34	39	36	right	2,3,4	none	n.a.
46	m	87	45	24	right, left	3,4; 3	TRHX	–
47	m	27	30	12	right	3	RETR	–
48	m	21	48	12	right	4,5	BTX, RTR, TRHX	–
49	m	34	34	3	right	3	BTX, TRHX	–
50	m	22	31	72	right	3,4,5	BTX	–
51	m	31	33	72	left	4	RTR	–
52	m	117	63	36	right	4,5	BTX	+
53	m	137	35	48	left	2	BTX, TRHX	n.a.
54	m	96	27	240	right, left	2,3,4; 3,4	TRHX	–
55	m	85	29	96	left	n.a.	TRHX	–
56	m	36	47	36	right	3	RTR	–
57	m	26	27	132	right	3,4	BTX, TRHX	–
58	m	59	18	480	left	1,2	BTX, RTR	n.a.
59	f	11	28	9	left	3,4,5	RTR	n.a.
60	m	44	28	12	right	2,3	BTX, TRHX	–
61	f	19	28	12	right	3	RTR	–
62	m	7	61	132	right	4,5	RTR	–
63	m	10	29	72	right	4,5	RTR	–
64	m	18	56	9	right	2	RTR	n.a.
65	m	39	42	60	left	4	RTR, TRHX	n.a.
66	f	14	26	30	left	2,4,5	BTX, RTR	–
67	m	50	39	12	right	2,3	BTX, RTR, TRHX	–
68	m	78	32	144	right	3,4	BTX	–
69	m	54	28	72	left	2,3,4,5	BTX, RTR, TRHX	–
70	m	22	44	6	right	4,5	RTR, TRHX	–
71	m	53	29	12	right	2,3,4	BTX, TRHX	n.a.
72	f	15	47	96	right	3,4	BTX, RTR, TRHX	n.a.
73	m	80	21	12	right	4,5	BTX	n.a.
74	m	31	36	6	left	3	BTX, RTR, TRHX	–
75	m	0	21	72	left	2	TRHX	+
76	f	47	n.a.	n.a.	right	1	TRHX	n.a.
77	m	184	33	24	right	3,4	TRHX	–
78	f	13	35	6	right	1,2,3	BTX, TRHX	–
79	f	25	34	12	right	2,4	BTX, RTR	–
80	m	5	41	60	right	3	RTR	–

*f* Female, *m* Male, *Treatment duration* Time between first and last video in months, Age first symptoms in years, *Time to treatment* Time between onset of symptoms and first appointment in months, *BTX* Botulinum toxin, *TRHX* Trihexyphenidyl, *RTR* Retraining, *n.a.* Data not available from patient files, + Positive family history of movement disorders, − Negative family history of movement disorders

### Video rating

Raters (*n* = 6, three female) were recruited from piano major classes at Hanover University of Music, Drama and Media. Student raters had previously attended a seminar on performing arts medicine which is part of the curriculum and held by the director of the institute and expert in MD (author EA), and were familiar with musician’s disorders such as focal dystonia. Furthermore, they were informed about general prevention and treatment options of this disorder and had seen videos of musician patients. They were psychologically stable and had never approached EA with personal issues of anxiety, psychological or medical problems. They received a two-hour introduction by EA, explaining again symptoms, risk factors and pathophysiology of MD. Subsequently, the rating process was thoroughly practiced with 12 example videos not included in the study. These “practice assessments” were compared and discussed in order to establish common criteria of rating concerning severity of symptoms. Lab-based rating was supervised by one of the authors (J.G.).

Raters evaluated scale-playing videos on 10 cm visual analogue scales (VAS) [25], with 0 and 10 as anchor points (see Additional file 1). We aimed to evaluate acoustic as well as visual aspects of scale playing with the following two criteria: Temporal regularity of notes (very regular to very irregular at both ends of the “acoustic” VAS) and motor impairment of the movement (not impaired to highly impaired at both ends of the “visual” VAS). For the rating of temporal regularity, raters were asked to evaluate the evenness of C-major scales by mainly paying attention to auditory information. For the rating of motor impairment, raters were instructed to identify the flexion of the dystonic finger(s) and the extension of the compensatory finger(s), a concept introduced by Candia et al. [26] and further developed by Spector and Brandfonbrener [22]. They were asked to rate the visual manifestation of these aberrant movements as an expression of muscular symptoms during scale playing.

For data analysis and presentation, we defined the VAS for irregularity such that high values represent very irregular and uneven scale playing. Raters provided a score for a video snippet with a vertical pencil stroke on the VAS line. A difference between two ratings in a given patient of, e.g. 1 cm on the VAS scale represented a difference of −1 or + 1 in the rating criteria.

Each video was rated twice by a pair of raters, resulting in four ratings per video. For 266 videos included in the study, this amounted to a total of 1064 ratings. The rating process was split up into several sessions to reduce fatigue in raters. The videos were equally and randomly assigned to the six raters, so that each rater did not rate every video, but approximately 177 videos in four sessions distributed over 4 days within 1 week, with 44 to 45 videos per session in randomized order.

A movement disorders expert (author EA) also rated the videos in order to be able to compare them to the student raters’ scores. Videos were again presented twice and in random order. In contrast to the student ratings however, the expert rating was not divided into clearly defined sessions for logistic reasons. Example videos from one patient included in the study are provided as additional file (see Additional files 2 and 3).

### Statistical analysis

Rating scores for each video were calculated as the average of each rater and averaged across the pair of raters, separately for irregularity and impairment. The rating scores of the MD-expert were not included in this analysis.

To assess intra- and inter-rater reliability of the students’ ratings, intra-class correlation (ICC) [27], average Pearson’s product-moment correlation and inter-rater concordance coefficient [28] were calculated. For an evaluation of the expert rating, we computed the average of the two ratings for each video. Again, intra-rater reliability with Pearson’s product-moment correlation and the inter-rater concordance coefficient were calculated to compare students and expert ratings.

To assess the temporal course of MD severity and the efficacy of the deployed therapies as well as the influence of patient-specific time factors (see below), we compared the rating scores of the first and last video available, omitting the ones in between, if any. By restricting the analysis to first and last videos we matched the number of observations per patient, considering that in 45% of the patients (*n* = 36) only two videos were available for the study. The omitted data will be presented elsewhere. Furthermore, for the main analysis we solely used the student ratings.

### Bayesian analysis of the data and creation of models

Data were subjected to Bayesian multilevel beta regression [29] with the irregularity and impairment rating scores of both videos as outcome after transformation of the original scores (between 0 and 10) to the range between 0 and 1. The outcome can be interpreted as ratio or percentage achieved of a possible maximum. Thus a (transformed) score of e.g. 0.65 is equivalent to 65% of the highest possible irregularity (or impairment). To assess the outcome at the two time points and to evaluate the influence of *time point* (first, last), *treatment* (retraining, Botulinum toxin injections, or Trihexyphenidyl medication) as well as *treatment duration* (in decades), *time to treatment* (time between first symptoms and treatment commencement; in decades) and *age at first symptoms* (in decades), increasingly complex regression models were built, starting with the Null model, containing only the individuals as grouping structure. Then, step by step, additional explanatory variables were added. Explanatory variables containing missing data (i.e., *time to treatment* and *age at first symptoms*) were modelled such that the missing values were estimated using the entire information available (see Additional files 4 and 5 for modelling formulae). Finally, the resulting models were compared to each other by means of leave-one-out cross-validation [30], and only the best models were reported, unless noted otherwise (see Additional files 6 and 7). Model priors are reported in Additional files 4 and 5. All continuous input variables were centered to ease computation and interpretation.

### Regression models

The best models with respect to expected log pointwise predictive density (elpd) [30] (see Additional files 6 and 7 for details on model comparison) for the outcomes irregularity and impairment contained in both cases no interactions for the population effects *time point* and *therapy*. Since this interaction was of special interest to answer the question of how large the impact of each therapy was over time on the two outcomes, we looked at those models with elpd values closest to the top model *and* that included an interaction term. For both outcomes, these were the models also containing treatment duration as a covariate (see Additional files 6 and 7). The elpd values of both these models were less than two standard errors away from the respective best model and it was therefore deemed acceptable to consider them.

## Results

### Rating reliability

Analysis of the intra-rater reliability of student ratings revealed a significant average Pearson’s product moment correlation both for irregularity and impairment of *r* = 0.84 (95%-confidence interval [0.81-0.86], *p* < 0.01) and a significant average ICC for irregularity of 0.86 (range: 0.83, 0.88) and for impairment of 0.88 (0.86, 0.90). The mean inter-rater concordance coefficient among students was *r* = 0.42 (0.05, 0.85) for irregularity ratings and *r* = 0.46 (0.14, 0.62) for impairment ratings. Intra-rater reliability was also significant in the expert rating, with correlation coefficients *r* = 0.7 ([0.64-0.76], *p* < 0.01) for irregularity and *r* = 0.78 ([0.73-0.82], *p* < 0.01) for impairment. The inter-rater concordance coefficient between students and expert was *r* = 0.43 [0.32-0.52] for irregularity and *r* = 0.48 [0.38-0.56] for impairment.

### Data exploration

**Fig. 1:**
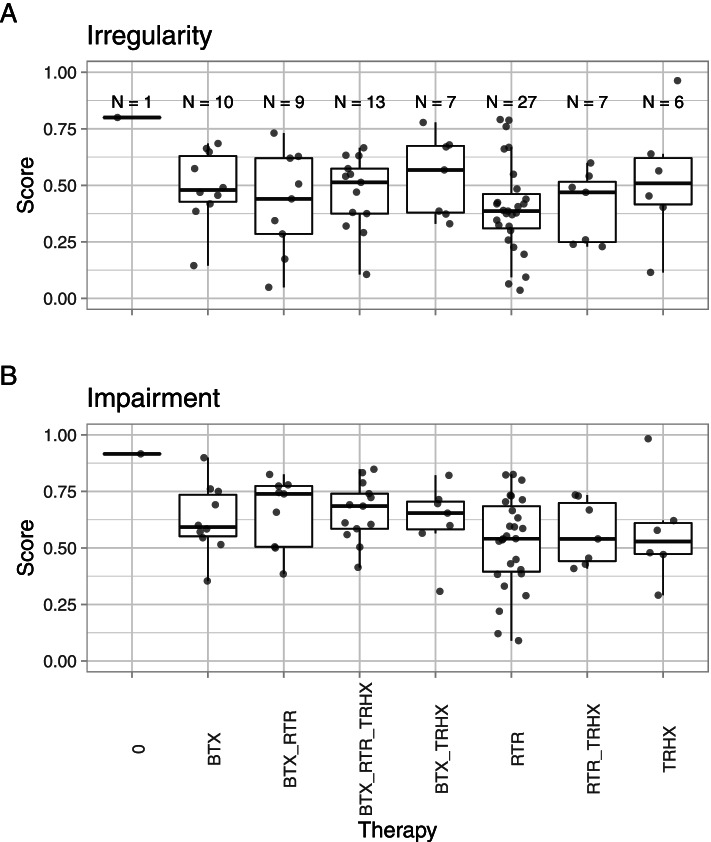
Raw data of mean rating scores before treatment onset for seven different therapies and therapy combinations along with one patient who received no treatment. Each dot represents the mean VAS score of two ratings provided by two raters each on a given video snippet. **A** Box-and-whisker plots of irregularity VAS-rating scores in the first video, i.e. before treatment commencement. **B** Box-and-whisker plots of impairment VAS-rating scores in the first video. Note: rating scores were divided by ten for analytical reasons. 0, one patient without treatment; See Table 1 for treatment abbreviations

Figure 1 shows that non-random assignment to therapies led to varying sample sizes per therapy group and substantially differing group medians in rating scores prior to treatment.

Patients who received retraining as a single therapy formed the largest group and had the lowest pre-treatment scores for both irregularity and impairment in impairment together with the combination of Retraining and Trihexyphenidyl as single therapy.

### Results of the regression models

The best models with respect to expected log pointwise predictive density (elpd) [24] (see Additional files 6 and 7 for details on model comparison) for the outcomes irregularity and impairment contained in both cases no interactions for the population effects *time point* and *therapy*. Since this interaction was of special interest to answer the question of how large the impact of each therapy was over time on the two outcomes, we looked at those models with elpd values closest to the top model *and* that included an interaction term. For both outcomes, these were the models also containing treatment duration as a covariate (see Additional files 6 and 7). The elpd values of both these models were less than two standard errors away from the respective best model and it was therefore deemed acceptable to consider them.

**Fig. 2:**
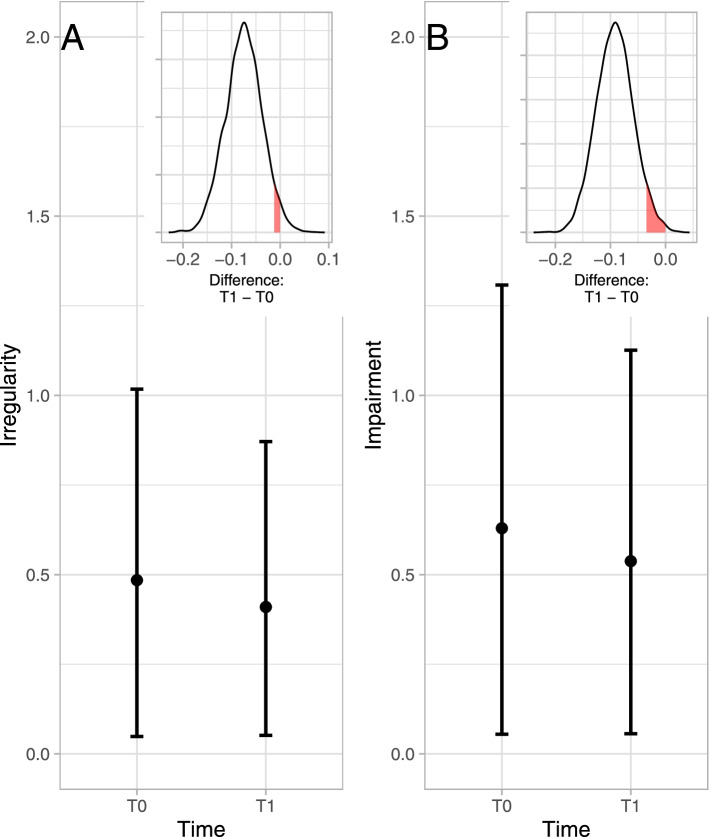
Estimated rating scores of irregularity (**A**) and impairment (**B**) at T0 (first video) and T1 (last video) across all therapies, with points at the respective mean of the posterior distributions, and error bars showing the 95% credibility interval. Insets present the differences between the posteriors at both time points, with the red shaded areas designating the region between the upper 5% of the density mass and zero, with zero corresponding to no difference between the two time points

Across all therapies average relative improvements of 14% in ratings of irregularity and 15% in ratings of impairment were estimated (see Fig. 2A, B).

The results of the best irregularity model containing an interaction term for time and therapy (model m4airr in Additional file 6) are presented in Table 2, whereas Table 3 shows the results of the best impairment model containing an interaction for time and therapy (model m4aimp in Additional file 7).

**Table 2:** Estimated mean irregularity scores at time points 0 and 1, the difference between the posterior distributions at both time points, the lower bound of the upper 95% of the difference distributions, and relative change between T0 and T1 for the eight therapy groups, as estimated by the best-fitting model with an interaction term (Therapy x Time). One patient (therapy: none) chose not to receive any treatment. See Table 1 for treatment abbreviations

Therapy	Time 0	Time 1	Difference	LB of upper 95%	Rel. change
None	0.580	0.487	−0.090	0.135	0.159
BTX	0.502	0.486	−0.015	0.129	0.032
BTX_RTR	0.403	0.423	0.0201	0.172	−0.049
BTX_RTR_TRHX	0.482	0.495	0.013	0.144	−0.026
BTX_TRHX	0.535	0.293	−0.237	−0.075	0.453
RTR	0.385	0.275	−0.10	−0.025	0.286
RTR_TRHX	0.407	0.350	−0.057	0.108	0.139
TRHX	0.583	0.459	−0.1245	0.056	0.213

**Table 3:** Estimated mean impairment scores at time points 0 and 1 along with the difference between the posterior distributions at both time points, the lower bound (LB) of the upper 95% of the difference distributions, and relative (rel.) change between T0 and T1 for the eight therapy groups, as estimated by the best-fitting model with an interaction term (Therapy x Time). One patient (therapy: none) chose not to receive any treatment. See Table 1 for treatment abbreviations

Therapy	Time 0	Time 1	Difference	LB of upper 95%	Rel. change
None	0.773	0.778	0.001	0.149	−0.007
BTX	0.637	0.571	−0.064	0.077	0.104
BTX_RTR	0.633	0.521	−0.111	0.039	0.176
BTX_RTR_TRHX	0.667	0.621	−0.046	0.068	0.069
BTX_TRHX	0.616	0.526	−0.089	0.081	0.146
RTR	0.500	0.387	−0.112	−0.022	0.226
RTR_TRHX	0.550	0.350	−0.196	−0.027	0.364
TRHX	0.679	0.561	−0.117	0.058	0.174

**Fig. 3:**
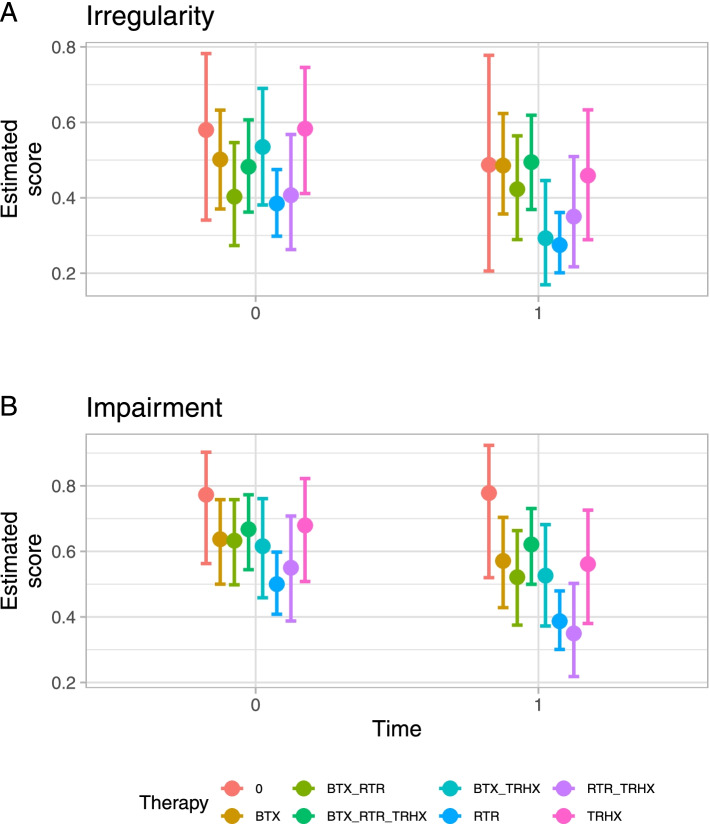
Average estimated scores (with 95% credibility intervals) in student ratings at T0 (first video) and T1 (last video); **A** irregularity, and **B** impairment. See Table 1 for treatment abbreviations

Only Botulinum toxin combined with Trihexyphenidyl (45% improvement), as well as retraining (29% improvement) alone yielded improvements such that 95% or more of the irregularity differences between T1 and T0 lay below zero; whereas in impairment, solely retraining alone (23% improvement) and retraining combined with Trihexiphenidyl (36% improvement) benefitted the patients to such a degree that 95% or more of the differences were located below zero (see Fig. 3A, B*)*.

## Discussion

This retrospective, observational study assessed the development of Musician’s Dystonia in 80 keyboard players, and its dependence on patient specific factors with a rating procedure of videos recorded over a period of almost 20 years. Patient cohort characteristics are similar to other studies on MD, whose epidemiologic data has been reviewed by Conti et al. [31]. The majority of patients were male, recognizing first symptoms of dystonia during their fourth decade of life. There was high variability in age at onset of symptoms, from early adulthood to retirement age. The right hand was predominantly affected and only a small number of patients presented symptoms in both hands. Our study has several limitations, due to its retrospective character and the fact that recording of videos took place during clinical examinations. Recordings were initially not intended to be used for a long-term study. Therefore, recording conditions were not standardized: no metronome was used, resulting in variable duration and tempo of scale playing; the camera angle with respect to the piano varied, with variable zoom settings. Furthermore, patients were not recorded at every visit, and recordings sometimes only took place when substantial changes of symptoms were apparent. Due to these circumstances, the number of recordings per patient and the time between these recordings differed substantially. We tried to minimize the effects of this heterogeneity by only analysing the first and last video in every patient. In addition, to adjust for varying follow-up durations we added the covariate “therapy duration” in our models.

The retrospective character of this study meant that therapies were heterogeneously and not always continuously used due to the individual needs and preferences of each patient. This led to varying sample sizes between the different therapy groups.

Considering these limitations, the main findings of the study however, are informative. First, video rating with piano students as a homogeneous rater group using the scales explained above was a useful tool to retrospectively rate symptoms of MD in pianists. According to the Dystonia Study Group, a sufficient rating scale for the evaluation of dystonia [32], adapted for MD [23, 33], needs to measure MD specifically, be sensitive to change, be reliable and valid, as well as efficient and practical in clinical use. Our rating procedure is highly specific for rating MD in pianists, by capturing the expression of symptoms (motor impairment of movement and temporal irregularity) during scale playing. The rating procedure used is also sensitive to change due to the use of visual analogue scales, which have been described as more sensitive in comparison to categorical Likert-Scales [34]. Furthermore, analyses of rating reliabilities showed strong intra-rater reliabilities and moderate inter-rater concordance in student and expert ratings (classification according to Schober et al. [35]). One reason for the lower inter-rater concordance might be the different interpretation of the two rating criteria by the raters. Although we performed an introductory session with detailed explanations and training, irregularity and impairment are still subjective criteria with possibilities for individual interpretation. Nonetheless, lower inter-rater concordance between the expert and students can be explained by the different clinical background and experience and, combined with the strong intra-rater reliability in student raters thus supports the idea of a homogeneous rater group. The decision to choose piano students as raters was motivated by their group being rather homogenous with regard to age, own playing expertise, exposure to judging others’ playing abilities as well as medical expertise in MD, which we tried to impart during the training session.

Second, the relative average improvement across all therapies was 14% in ratings of irregularity and 15% in ratings of impairment, corresponding to an average change of − 0.74 cm and − 0.92 cm, respectively, on the visual analog scale. With the deployed methods we were able to objectively verify a moderate improvement of MD symptoms in pianists over time. Van Vugt et al. also reported a tendency towards more regular scale playing in objective MIDI-analyses in pianists [17]. Although the methods to calculate improvement rates differed, our study provides further evidence suggesting that therapy in MD in pianist has an objectively measurable positive effect on patients’ playing ability. Considering the fact that the data in our study and in the study by Van Vugt et al. were collected from overlapping time windows and from the same patient sample opens new possibilities for further studies by comparing the two data sets. Furthermore, it is noteworthy that ratios of quantitative improvement in objective rating in our study are substantially different from those in subjective rating in previous studies (e.g. 31% improvement in patient subjective questionnaires [17]). Van Vugt et al. stated the hypothesis that patients had the tendency to overestimate in a positive way the development of symptoms. This hypothesis can be supported by findings of Lee et al. reporting no difference in life satisfaction of patients with MD and healthy musicians. They assumed that patients with MD find a way to cope with dystonia regardless of the course of symptoms [36].

Third, therapies seem to show differing effects on treatment outcome. While Botulinum toxin in combination with Trihexyphenidyl showed the highest relative improvement in irregularity (45%, corresponding to an average improvement of 0.75 cm; Table 2), the combination of retraining and Trihexyphenidyl showed the highest improvement in impairment (36%, equivalent to a change of − 1.7 cm; Table 3). The outcome of those patients receiving a combination of therapies has to be interpreted carefully though, considering the relatively small number of patients in the respective groups and the retrospective, observational design of this study as explained above. Unsuitable therapies were discontinued while other therapies were started in the treatment process for patients with an insufficient outcome in one therapy. For example, patients who chose to receive all three therapies might be characterized by a generally low treatment response and were thus possibly trying all available options, which in turn may explain the low effect on treatment outcome in the group BTX + RTR + TRHX. However, one might be surprised by the comparatively small effect of Botulinum toxin treatment alone (3 and 10% relative improvement in irregularity and impairment, respectively), contradicting self-report data by Schuele et al. which indicated a mild to marked improvement after therapy with Botulinum toxin in 69% of patients with MD [13]. The low relative average improvement rate in our study might be explained by the fact that video recording took place during regular clinical examination and patients receiving injections with Botulinum toxin mostly asked for clinical appointments after deterioration of symptoms with the need for a new injection.

The fact that retraining was the most effective therapy fits into the results of previous studies comparing treatment effectiveness with subjective patient questionnaires. Van Vugt et al. reported that self-ratings attributed retraining a significantly stronger effect than Trihexyphenidyl, and Jabusch et al. reported an improvement in 50% of patients taking part in pedagogical retraining [11, 17]. It should be mentioned that due to the adaptive use of therapies, driven by personal preferences and needs of the patients we could not extract factors predicting the efficacy of specific treatments in this retrospective analysis. According to our impression, − which has yet to be proven scientifically -, rather personality factors than phenotype of dystonia and type of therapy predicted a more positive outcome. Openness to new experiences, inventiveness of coping strategies, flexibility of repertoire choice, a sense of self-efficacy and an optimistic attitude with caring personal relations seem to be essential for a “good live” with MD.

## Conclusions

In conclusion this study provides a new perspective on the course of Musician’s Dystonia in keyboard players. The applied rating method showed sufficient intra-rater reliability and was efficient and useful by allowing rating of many patients in simple way, due to its intuitive use and the small number of rating criteria. We propose its use for a fast overview during regular clinical examination, though further validation is needed. We have shown positive treatment effects for all three commonly used therapeutic approaches, with clearest improvement following retraining therapy.

## Supplementary Information

**Additional file 1.** Visual analogue rating scale (VAS). VAS rating scale used in the rating process. Two rating criteria were assessed (temporal regularity and motor impairment of the movement). Raters had to fill in the displayed video number and mark their rating with a vertical line.

**Additional file 2.** First examination 2002. Pianist with focal dystonia in right hand during first examination in 2002. Rating of Irregularity: 7.6, rating of Impairment: 8.25.

**Additional file 3.** Second examination 2006. Pianist with focal dystonia in right hand during last examination in 2006. Treatment with Retraining. Rating of irregularity: 2.29, rating of impairment: 2.46.

**Additional file 4.** Informative priors placed on parameter estimates for model m4airr (see Additional file 6).

**Additional file 5.** Informative priors placed on parameter estimates for model m4aimp (see Additional file 7).

**Additional file 6. **Leave-one-out cross-validation estimates (via expected log pointwise predictive density, elpd) of regression models estimating dystonia-related irregularity while playing C-major scales on the piano keyboard. The model with the largest elpd is deemed to be the best model and listed at the top, and the other models are listed by decreasing epld, with the column elpd_diff showing their ‘distance’ to the top model. The column *se_diff* contains the pairwise standard error of each model with the top model. If a model lies more than approximately two SE away from the top model it is considered to be meaningfully worse.

**Additional file 7. **Leave-one-out cross-validation estimates (via expected log pointwise predictive density, elpd) of regression models estimating dystonia-related movement impairment while playing c-major scales on the piano keyboard. The model with the largest elpd is deemed to be the best model and listed at the top, and the other models are listed by decreasing epld, with the column elpd_diff showing their ‘distance’ to the top model. The column *se_diff* contains the pairwise standard error of each model with the top model. If a model lies more than approximately two SE away from the top model it is considered to be meaningfully worse.

## Data Availability

The videos, data and statistical algorithms are fully available in the Institute of Music Physiology and Musician’s Medicine of the University of Music, Drama and Media upon request.

## Abbreviations

BTX: Botulinum toxin
EMG: Electromyography
MD: Musician’s Dystonia
ICC: Intra-class correlation
RTR: Retraining
TRHX: Trihexyphenidyl
VAS: Visual Analogue Scale
