# Supplementary figures and images for: Video-based long-term follow up of musician’s dystonia in pianists reveals similar improvements following different treatment strategies: a retrospective observational study
Source: J Clin Mov Disord. 2022 Jan 25;7(Suppl 1):10. doi: 10.1186/s40734-021-00092-3 (PMC8787894; doi:10.1186/s40734-021-00092-3)

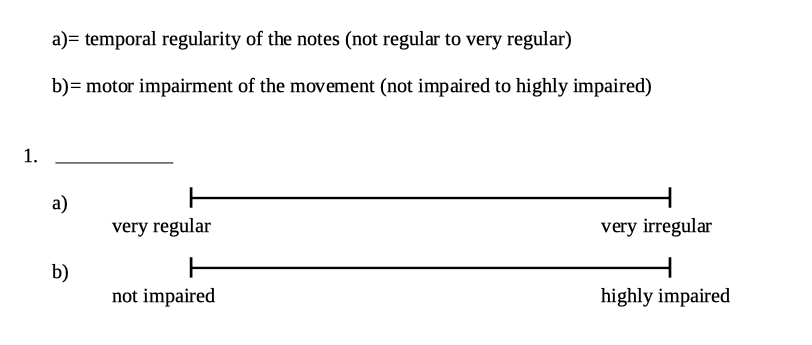

Supplement: Supplementary file 1 — Additional file 1. Visual analogue rating scale (VAS). VAS rating scale used in the rating process. Two rating criteria were assessed (temporal regularity and motor impairment of the movement). Raters had to fill in the displayed video number and mark their rating with a vertical line. [file 40734_2021_92_MOESM1_ESM.png]
